# Exhausted Woods from Tannin Extraction as an Unexplored Waste Biomass: Evaluation of the Antioxidant and Pollutant Adsorption Properties and Activating Effects of Hydrolytic Treatments

**DOI:** 10.3390/antiox8040084

**Published:** 2019-04-01

**Authors:** Lucia Panzella, Federica Moccia, Maria Toscanesi, Marco Trifuoggi, Samuele Giovando, Alessandra Napolitano

**Affiliations:** 1 Department of Chemical Sciences, University of Naples “Federico II”, Via Cintia 4, I-80126 Naples, Italy; federica.moccia@unina.it (F.M.); maria.toscanesi@unina.it (M.T.); marco.trifuoggi@unina.it (M.T.); alesnapo@unina.it (A.N.); 2 Centro Ricerche per la Chimica Fine Srl for Silvateam Spa, Via Torre 7, 12080 San Michele Mondovì, CN, Italy; sgiovando@silvateam.com

**Keywords:** agri-food waste, exhausted wood, antioxidant, DPPH assay, FRAP assay, tannins, heavy metals, methylene blue, nitric oxides, acid hydrolysis

## Abstract

Exhausted woods represent a byproduct of tannin industrial production processes and their possible exploitation as a source of antioxidant compounds has remained virtually unexplored. We herein report the characterization of the antioxidant and other properties of practical interest of exhausted chestnut wood and quebracho wood, together with those of a chestnut wood fiber, produced from steamed exhausted chestnut wood. 2,2-Diphenyl-1-picrylhydrazyl (DPPH) and ferric reducing/antioxidant power (FRAP) assays indicated good antioxidant properties for all the materials investigated, with exhausted chestnut wood, and, even more, chestnut wood fiber exhibiting the highest activity. High efficiency was observed also in the superoxide scavenging assay. An increase of the antioxidant potency was observed for both exhausted woods and chestnut wood fiber following activation by hydrolytic treatment, with an up to three-fold lowering of the EC_50_ values in the DPPH assay. On the other hand, exhausted quebracho wood was particularly effective as a nitrogen oxides (NO_x_) scavenger. The three materials proved able to adsorb methylene blue chosen as a model of organic pollutant and to remove highly toxic heavy metal ions like cadmium from aqueous solutions, with increase of the activity following the hydrolytic activation. These results open new perspectives toward the exploitation of exhausted woods as antioxidants, e.g., for active packaging, or as components of filtering membranes for remediation of polluted waters.

## 1. Introduction

In the light of their marked antioxidant properties, phenolic polymers from natural sources have been the focus of increasing interest as sustainable functional materials for the wide range of potential applications, for example as biocompatible materials for biomedical devices or as active components in functional food packaging [1,2,3,4,5,6,7]. Indeed, phenolic polymers display manifold advantages over their monomers, including higher stability properties thus offering easier handling and processing, as well as lower solubility reducing tendency to be released [4,8,9].

**Figure 1:**
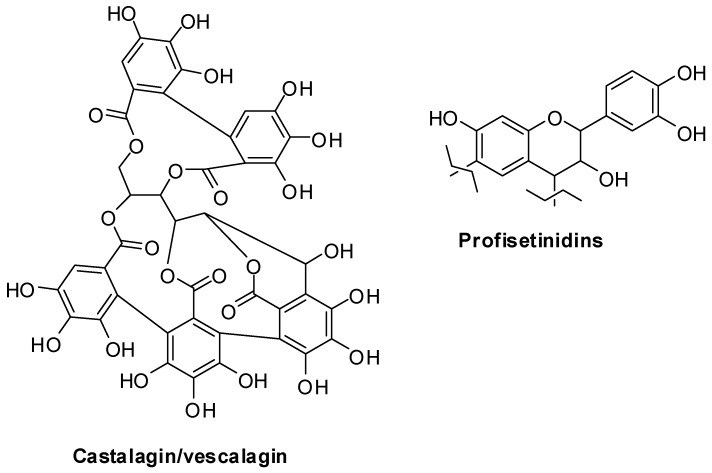
Representative structures of the main tannins occurring in chestnut and quebracho wood.

Among natural phenolic polymers, tannins occupy a prime position given the well-established antimicrobial and protein binding properties [1,10,11,12,13,14]. Tannins are traditionally ranked into the hydrolyzable and the condensed or non-hydrolyzable classes. *Castanea sativa* wood extract, commonly known as chestnut tannin, is composed mainly of hydrolyzable ellagitannins such as castalagin and its isomer vescalagin, whereas quebracho tannin, extracted from the hardwood of *Shinopsis balansae/lorentzii*, comprise mainly condensed tannins of which linear profisetinidins represent the major constituents (Figure 1) [1,15,16].

Both chestnut and quebracho tannins are commonly employed not only in the tanning industry, but also in the oenological, cosmetic, and pharmaceutical field as well as in products for animal feed [1,17,18,19,20,21,22,23]. Tannin extraction from wood is generally performed in hot water. The residual wood biomasses (exhausted woods), a by-product of the tannin extraction process, may be taken as renewable sources in their own right, since the wood has been treated only with hot water at high pressure, a treatment that is not expected to result in any significant change of the structural components. These exhausted woods are commonly used in the production of pellets for heating and energy production. The isolation of cellulose nanocrystals to be used e.g., as nanofillers for polymer composites from the exhausted acacia bark, obtained after the industrial process of extracting tannin, has also been reported [24]. However, the possible exploitation as antioxidant materials has remained virtually unexplored.

**Figure 2:**
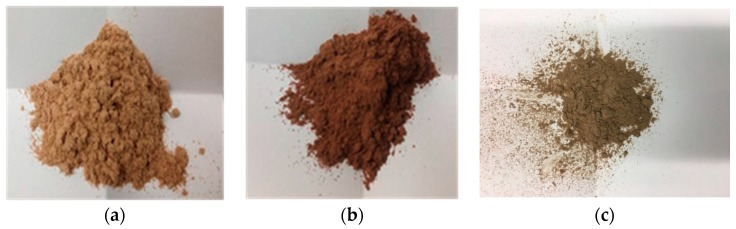
(**a**) Exhausted chestnut wood; (**b**) exhausted quebracho wood; (**c**) chestnut wood fiber.

We report herein the characterization of the antioxidant properties of exhausted chestnut and quebracho wood, together with those of a chestnut wood fiber produced from steamed exhausted chestnut wood. The materials investigated are shown in Figure 2. For comparison, the corresponding fresh woods and tannins were investigated as well. Exhausted woods and chestnut wood fiber were also tested for their ability to remove environmental pollutants, either organic compounds or toxic heavy metals. Based on recent findings showing that acid hydrolytic treatment of natural phenolic polymers or wastes leads to materials with potent antioxidant efficiency [4,25,26], we also investigated the properties of the exhausted woods and chestnut wood fiber materials obtained by such treatment in comparison with those of the untreated materials, showing in most cases a significant enhancement of the activity. The results obtained open new perspectives for the exploitation of these by-products e.g., in active packaging or for remediation of polluted waters.

## 2. Materials and Methods

### 2.1. General Experimental Methods

Chestnut and quebracho exhausted and fresh woods, chestnut wood fiber as well as chestnut and quebracho tannins were provided by Silvateam (Via Torre, S. Michele Mondovì, Cuneo, Italy). In particular, fresh chestnut and quebracho woods were obtained from hardwood of *Castanea sativa* and *Schinopsis lorentzii* respectively, and reduced in chips of ca. 1–3 cm. Tannins were obtained by soaking the wood chips in autoclaves with water, at 120 °C, under pressure; the extracts thus obtained were concentrated with a multiple-effect evaporator under vacuum until ca. 50% water was removed, and tannin powder finally obtained by spray-drying. Residual chips after the extraction were stored as exhausted woods. Chestnut wood fiber was obtained from chestnut exhausted wood after drying in oven overnight at 60 °C and milling to obtain <250 μm particles.

2,2-Diphenyl-1-picrylhydrazyl (DPPH), iron (III) chloride (97%), 2,4,6-tris(2-pirydyl)-*s*-triazine (TPTZ) (≥98%), (±)-6-hydroxy-2,5,7,8-tetramethylchromane-2-carboxylic acid (Trolox) (97%), ethylenediaminetetraacetic acid (EDTA) (>99%), nitroblue tetrazolium (NBT) chloride (98%), pyrogallol (≥98%), quercetin (≥95%), fluorescein, 2,2′-azobis(2-methylpropionamidine) dihydrochloride (AAPH) (97%), Folin & Ciocalteu’s phenol reagent (2N), gallic acid (≥97.5%), activated carbon, sodium nitrite (≥97.0%), *N*-(1-naphthyl)ethylenediamine dihydrochloride (≥98%), sulfanilamide (≥99%), methylene blue (MB), cadmium carbonate (99%), and nitric acid (≥69% v/v, TraceSELECT^®^ water solution) were obtained from Sigma-Aldrich (Milan, Italy) and used as obtained.

UV-Vis spectra were performed using a HewlettPackard 8453 Agilent spectrophotometer.

Fluorescence spectra were record on a HORIBA Jobin Yvon Inc. FluoroMax^®^-4 spectrofluorometer.

For metal removal experiments, 1% HNO_3_, 0.1 M HCl and 0.01 M phosphate buffer (pH 7.0) were prepared using ultrapure deionized water with conductivity <0.06 μS/cm. All glassware used were carefully washed first with a 1% HNO_3_ solution and then with ultrapure deionized water. Metal analysis was carried out on an inductively coupled plasma mass spectrometry (ICP-MS) instrument Aurora M90 model by Bruker.

### 2.2. Hydrolytic Treatment

The proper material (3 g) was treated with 70 mL of 6 M HCl under stirring at 100 °C overnight [4,26]. After cooling at room temperature, the mixture was centrifuged (8247× *g*, room temperature, 15 min) and the precipitate washed with water until neutrality and freeze dried. The recovery yields were 58% (chestnut wood fiber), 68% (exhausted chestnut wood) and 83% (quebracho exhausted wood).

### 2.3. DPPH Assay

To a 0.2 mM methanolic solution of DPPH wood or tannin powders were added (final dose 0.03–1.5 mg/mL) and after 10 min under stirring at room temperature the absorbance of the solution at 515 nm was measured [27,28]. Experiments were run in triplicate. Trolox was used as reference antioxidant. 

### 2.4. Ferric Reducing/Antioxidant Power (FRAP) Assay

To 0.3 M acetate buffer (pH 3.6) containing 1.7 mM FeCl_3_ and 0.83 mM TPTZ, wood or tannin powders (final dose 0.00625–0.3 mg/mL) were added and after 10 min under stirring at room temperature the absorbance of the solution at 593 nm was measured [29]. Results were expressed as Trolox equivalents. Experiments were run in triplicate.

### 2.5. Superoxide Scavenging Assay 

Wood or tannin powders (final dose 0.0625 mg/mL) were added to 0.05 M ammonium hydrogen carbonate buffer (pH 9.3) containing 0.4 mM EDTA and 12 μM NBT, followed by a 20 mM pyrogallol solution in 0.05 mM HCl (final pyrogallol concentration 3.3 mM) [4,30]. The mixture was vigorously stirred for 5 min, after that absorbance at 596 nm was measured. Results were expressed as percentage of reduction of the absorbance at 596 nm of a control mixture run in the absence of sample. Experiments were run in triplicate.

### 2.6. Oxygen Radical Antioxidant Capacity (ORAC Assay)

Wood or tannin powders (final dose 0.075 mg/mL) were incubated in a 62.7 μM fluorescein solution in 75 mM phosphate buffer (pH 7.4) for 30 min, at 37 °C. A 153 mM AAPH solution was then added (final APPH concentration 19 mM) and after stirring at 37 °C for 45 min fluorescence was measured (λ_ex_ = 485 nm, λ_em_ = 511 nm) [31]. Results were expressed as relative fluorescence intensity with respect to a control mixture run in the absence of APPH. Experiments were run in triplicate.

### 2.7. MB Adsorption Assay

Adsorption experiments were performed at room temperature by adding wood samples (0.2 mg/mL) to a 5 or 25 mg/L aqueous solution of MB. The mixtures were taken under stirring and after 30 min the absorbance at 654 nm was measured [32]. Activated carbon was used as reference material. Experiments were run in triplicate. 

### 2.8. Nitric oxides (NO_x_) Scavenging Assay

A solution of sodium nitrite (1 M) in water was added to 10% sulfuric acid over 10 min [33]. The red orange gas that developed (0.2–0.6 mL) was withdrawn with a syringe and conveyed through a tip containing 5 mg of wood sample into 3 mL of Griess reagent (0.5% sulfanilamide and 0.05% *N*-(1-naphthyl)ethylenediamine dihydrochloride in 1.25% phosphoric acid) and the absorbance at 540 nm was measured. Experiments were run in triplicate.

### 2.9. Cd^2+^ Adsorption

A 1.5 mM stock solution of the heavy metal was prepared by dissolving 10 mg of cadmium carbonate in 39 mL of 0.1 M HCl. Prior to the adsorption experiments a 1.5 mg/mL suspension of each wood sample in 0.01 M phosphate buffer (pH 7.0) was obtained by homogenization in a Tenbroeck glass to glass homogenizer for 4 min. 0.7 mL of the wood suspensions and 0.1 mL of the metal solution were added to 10 mL of 0.01 M phosphate buffer at pH 7.0. After 2 h, the mixtures were filtered through a 0.45 μm nylon membrane, acidified by addition of 69% nitric acid (1:100 *v*/*v*), properly diluted with 1% nitric acid, and analyzed by ICP-MS [34]. A calibration curve was built with cadmium solutions at five different concentrations. For each binding experiment a blank experiment was planned in which the metal ion was added in the phosphate buffer and incubated for 2 h without addition of the wood sample. Experiments were run in triplicate.

### 2.10. Evaluation of the Solubility of Woods and Tannins in the Assay Media 

Wood or tannin samples (3 mg) were added to methanol (20 mL), 0.3 M acetate buffer (pH 3.6) (20 mL), or water (15 mL), and taken under magnetic stirring. After 10 or 30 min the supernatants obtained after centrifugation (8247× *g*, room temperature, 15 min) were analyzed by UV-Vis spectrophotometry.

### 2.11. Determination of the Amount of Tannins in the Wood Samples 

Wood or tannin samples (10 mg) were stirred in 1 mL of a 1:1 *v*/*v* acetone/water mixture containing 1% acetic acid [35]. After 60 min the supernatants obtained after centrifugation (3534× *g*, room temperature, 20 min) were analyzed by UV-Vis spectrophotometry after 1:500 *v*/*v* dilution in methanol. The amount of tannins in each wood sample was determined by comparison of the absorbance at 269 nm (chestnut-derived samples) or 280 nm (quebracho-derived samples) with that measured for chestnut or quebracho tannins, respectively.

### 2.12. Measurement of Total Phenolic Content (TPC) 

Wood or tannin samples (10 mg) were stirred in DMSO (1 mL) for 1 h. After centrifugation (3534× *g*, room temperature, 20 min) 1–50 μL of the supernatant were added to 1.4 mL of water followed by 0.3 mL of a 75 g/L Na_2_CO_3_ solution and 0.1 mL of Folin & Ciocalteu’s reagent. After 30 min incubation at 40 °C, absorbance at 765 nm was measured [36]. Gallic acid was used as reference compound. Experiments were run in triplicate.

## 3. Results and Discussion

### 3.1. Antioxidant Properties of Exhausted Woods

In a first series of experiments the antioxidant properties of chestnut wood fiber and exhausted chestnut and quebracho woods were investigated with respect to the corresponding fresh woods and tannins by widely used assays, i.e. DPPH, FRAP, superoxide scavenging, and ORAC assays following the “QUENCHER” method which allows one to measure the efficiency of electron transfer processes from a solid antioxidant [27,28,29,30,31].

#### 3.1.1. DPPH and FRAP Assays

Table 1 reports the EC_50_ value, which is the dose of the material at which a 50% DPPH reduction is observed, determined for tannins and wood samples in the DPPH assay. For comparison data for the reference antioxidant Trolox are also reported.

Among the waste materials, chestnut wood fiber displayed the most promising DPPH-reducing ability, with an EC_50_ value of 0.054 mg/mL that compares well with that of Trolox (3.6-fold higher). Also quebracho and particularly chestnut exhausted wood exhibited quite low EC_50_ values, much lower than those reported for other agro-food wastes, such as spent coffee grounds (EC_50_ = 5.00 mg/mL) [4]. As expected, higher antioxidant activities were exhibited by fresh wood samples, still containing tannins, and tannins themselves, characterized by EC_50_ values approaching that of Trolox.

**Figure 3:**
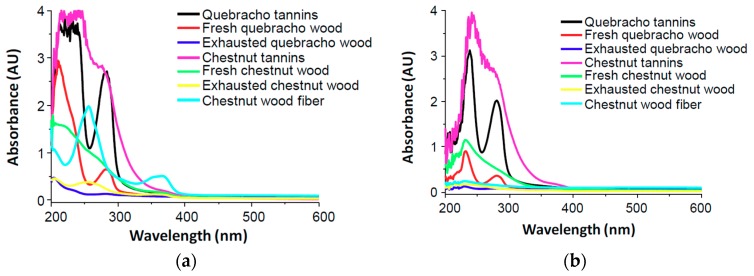
UV-Vis spectra of wood and tannin samples (0.15 mg/mL) in methanol (**a**) or 0.3 M acetate buffer (pH = 3.6) (**b**).

The marked differences observed in the antioxidant activity may be interpreted considering the solubility of the materials in the assay medium. Spectra shown in Figure 3a clearly indicate release of UV absorbing species in the case of pure tannins, chestnut wood fiber, and fresh woods, whereas exhausted woods do not give rise to appreciable absorbance.

The results of the FRAP assay (Table 1) looked less encouraging than those obtained in the DPPH assay, with all the waste materials exhibiting an iron(III)-reducing activity far lower than Trolox (Trolox equivalents <<1). Only chestnut tannins showed a satisfactory antioxidant power in this assay, whereas the fresh wood samples as well as quebracho tannins performed much less. Here again the antioxidant activity parallels fairly well the solubility in the medium used for the FRAP assay (Figure 3b).

**Figure 4:**
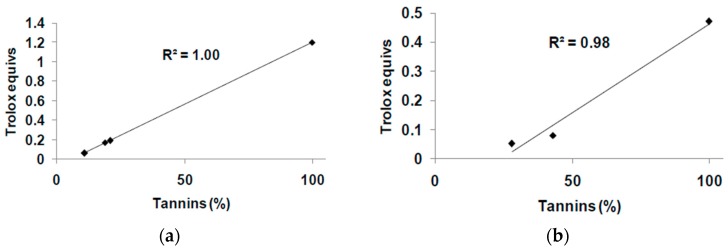
Correlation between tannin content and Fe^3+^-reducing activity of tannin and wood samples. (**a**) Chestnut-derived samples; (**b**) Quebracho-derived samples.

To obtain information about the compounds responsible for the antioxidant properties observed, the amounts of tannins and the TPC were determined for each wood sample (Table 2). As shown in Figure 4, a good linear correlation was found between Trolox equivalents determined in the FRAP assay and the tannin content (for both the hydrolyzable and non-hydrolyzable class), but not TPC (*R*^2^ = 0.59), pointing to residual tannins as the main determinants of the iron-reducing properties. On the contrary, DPPH reducing ability was not apparently related to either TPC (*R*^2^ = 0.37) or tannin content (*R*^2^ = 0.33 and 0.50 for chestnut and quebracho-derived samples, respectively), suggesting that several factors (e.g., relative solubility in the assay medium) might be involved in the different activities observed.

**Figure 5:**
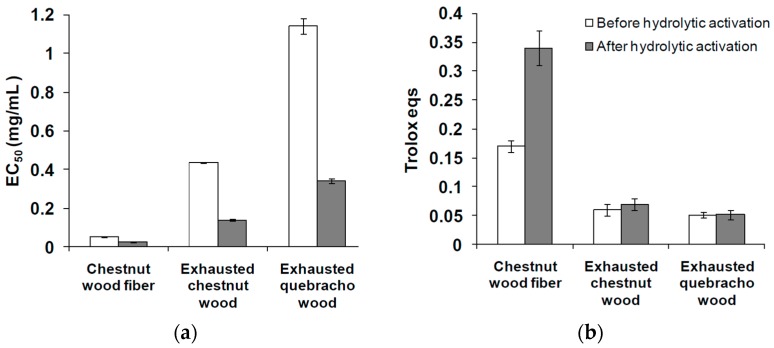
Antioxidant properties of exhausted wood samples before and after hydrolytic activation. (**a**) DPPH assay; (**b**) ferric reducing/antioxidant power (FRAP) assay. Reported are the mean ± SD values of at least three experiments.

The exhausted woods and the chestnut wood fiber that have been subjected to the hydrolytic treatment according to the protocol developed in previous studies [4,25,26] were then evaluated for their antioxidant activity by the DPPH and FRAP assays. Data shown in Figure 5a indicate for both exhausted quebracho and chestnut woods a decrease of EC_50_ values in the range of 25%–30% compared to the values obtained for the untreated materials. By contrast no significant increase of the activity was observed in the FRAP assay following the hydrolytic treatment, apart for chestnut wood fiber, which exhibited a two-fold increase in Trolox eqs (Figure 5b).

#### 3.1.2. Superoxide Scavenging Assay

**Figure 6:**
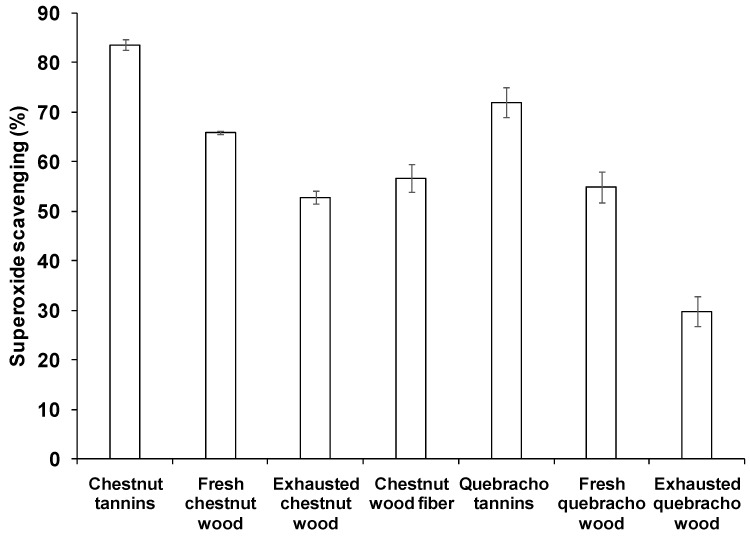
Superoxide scavenging activity of tannins and wood samples. Reported are the mean ± SD values of at least three experiments.

A very high efficiency, compared to other agri-food waste products such as spent coffee grounds [4], was observed in the superoxide scavenging assay (Figure 6), with tannins and fresh woods being always the most active samples, although in the case of chestnut-derived materials both the exhausted woods exhibited an activity comparable to that of the native sample. Only a modest correlation (*R*^2^ = 0.90 and 0.82 for chestnut and quebracho-derived samples, respectively) was found between the percentage of superoxide scavenging and the tannin content, whereas no correlation was found with TCP. Moreover, no significant improvement in the scavenging ability was detected in exhausted woods subjected to the hydrolytic treatment.

#### 3.1.3. ORAC Assay

The relative fluorescence intensities determined in the ORAC assay are reported in Table 3. In this case major differences were apparent between tannins/fresh woods and exhausted samples, with the first ones almost totally inhibiting fluorescein oxidation and the second ones being completely inactive, with the only exception of chestnut wood fiber, which showed an activity comparable to that of fresh chestnut wood. Notably, in this case a good linear correlation (*R*^2^ = 0.95) was found between the antioxidant activity of the wood samples and TPC; on the contrary no significant correlation was found with the amount of residual tannins. No effect of the hydrolytic treatment was apparent either in this assay.

The results of the antioxidant assays further add to the potential of wood fiber as a reinforcement in polymers [37,38] or as an ecological-friendly medium for horticultural practice to increase the antioxidant activity of fruit and vegetables [39].

### 3.2. Pollutant Adsorption Properties of Exhausted Woods

In a further series of experiments the adsorption capacity of the exhausted woods toward various environmental pollutants was evaluated. These included MB, as a model organic dye, NO_x_, that is nitric oxide (NO) and nitrogen dioxide (NO_2_) which are reactive nitrogen species commonly present in cigarette smoke and exhaust gases able to induce oxidative and nitrosative stress in humans, and cadmium ions (Cd^2+^), as a model of toxic heavy metals.

#### 3.2.1. MB Adsorption Assay

Table 4 reports the percentages of MB adsorption by the wood samples.

**Figure 7:**
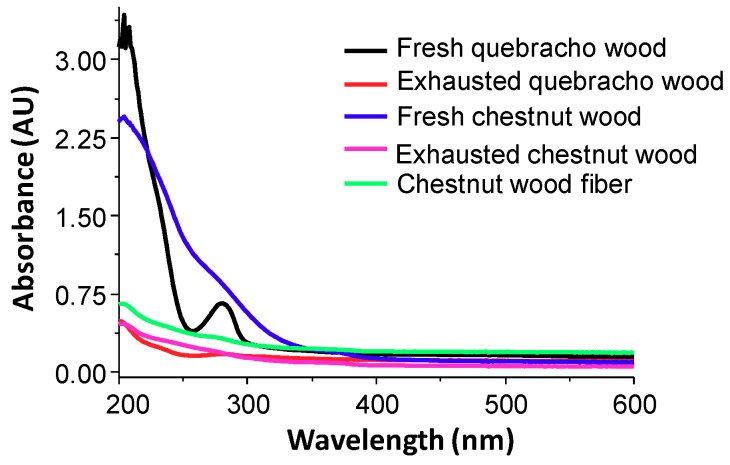
UV-Vis spectra of wood samples (0.2 mg/mL) in water.

Both exhausted chestnut and quebracho woods proved to be very efficient at a dose of 0.2 mg/mL, and their adsorption properties were comparable to those exhibited by the fresh wood samples. Indeed, UV-Vis spectrophotometric analysis (Figure 7) indicated a lower solubility in the assay medium (water) of chestnut wood fiber and exhausted woods compared to the fresh samples, a feature that further adds to the potential of these materials for removal of organic compounds from waste waters.

**Figure 8:**
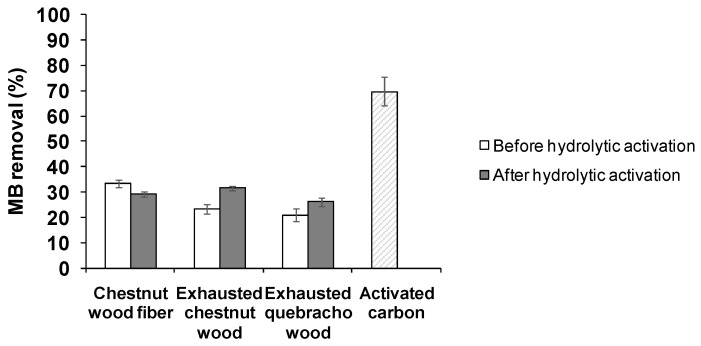
MB adsorption by chestnut wood fiber and exhausted woods before and after hydrolytic activation (MB starting concentration 25 mg/L). Reported are the mean ± SD values of at least three experiments. For comparison data relative to activated carbon are also reported.

A 20%–30% dye removal was still observed when wood samples were added to a more concentrated MB solution (25 mg/mL) (Figure 8). For comparison, under the same conditions a 70% MB removal was obtained with activated carbon, taken as a reference material. In contrast to what observed in the antioxidant assays, the MB adsorption ability of the exhausted woods did not change appreciably following to acid treatment.

#### 3.2.2. NO_x_ Scavenging Assay

All the samples examined led to >70% scavenging when 0.2 mL of NO_x_ gases were passed through 5 mg of wood (data not shown). The percentages of NO_x_ scavenging determined in the experiments run with 0.6 mL of gas are reported in Table 4, from which the superior ability of quebracho woods stands out compared to chestnut woods. These data would suggest a higher trapping efficiency of condensed tannins with respect to hydrolyzable tannins that could likely be ascribed to the reactivity of the resorcinol moieties of profisetinidins toward electrophiles like nitric oxides [40]. As observed above for MB adsorption, also in this case fresh woods were only slightly more active than exhausted samples. Notably, further to acid treatment a lower efficiency was observed for most of the tested woods (not shown), pointing to a role of the hydrolyzable cellulosic matrix in NO_x_ scavenging.

#### 3.2.3. Cd^2+^ Removal Assay

In a last series of experiments the ability of wood samples to remove heavy metals from aqueous solutions was investigated using Cd^2+^ as model ions. The assay was performed at pH 7.0, based on previous observations showing that natural phenolic polymers do not exhibit significant chelating properties at acidic pH [34]. In this case fresh woods were not evaluated because of the release of significant amounts of material under the testing conditions, likely due to solubilization of residual tannins and/or other low molecular weight components.

**Figure 9:**
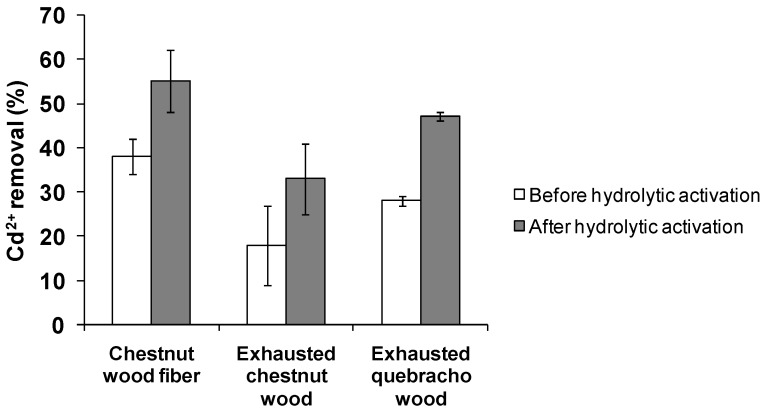
Cd^2+^ removal by exhausted woods before and after hydrolytic activation. Reported are the mean ± SD values of at least three experiments.

Based on the data reported in Figure 9, chestnut wood fiber was again the most active among the waste materials, followed by exhausted quebracho wood. An increase in Cd^2+^ removal was observed following hydrolytic activation for all the woods examined.

## 4. Conclusions

Exhausted woods represent a largely available waste product of the tannin extraction process, whose practical exploitation has not yet been duly considered. As reported in the present paper, these materials exhibit good antioxidant properties in the DPPH and superoxide scavenging assay, and are also able to efficiently adsorb pollutants such as toxic gases, organic dyes and heavy metals. In particular, chestnut woods, containing mainly hydrolyzable tannins, were particularly active as antioxidants, whereas quebracho woods, characterized by the presence of condensed tannins, were found to be more effective as adsorbent materials, especially toward NO_x_ fumes. Of particular interest is the enhancement of the antioxidant properties of exhausted woods and chestnut wood fiber following hydrolytic treatment, a methodology that had already been applied to materials containing lignins like spent coffee grounds [4] and the black sesame seed pigment [25], but also ellagitannins like pomegranate wastes [26]. An interpretation of such effects at a molecular level invoke aromatization and dehydration processes enhancing the hydrogen donor ability of the OH functionalities stemming from delocalization of the resulting phenoxyl radical over a more conjugated molecular backbone [4,8]. On the other hand, from consideration of the moderate weight loss further to the hydrolytic treatment it cannot be argued that the observed activation effects may simply be ascribed to removal of cellulosic or other inactive components of the materials.

Overall, these results would point to exhausted woods as novel potential functional additives to be used for example in active packaging, as food stabilizers against oxidative deterioration, or in filtering devices as pollutant removal agents.

## Figures and Tables

**Table 1:** Antioxidant properties of tannins and wood samples.^1^

Sample	EC_50_ (mg/mL) ^2^ (DPPH Assay)	Trolox Equivalents (FRAP Assay)
Chestnut wood fiber	0.054 ± 0.003	0.17 ± 0.01
Exhausted chestnut wood	0.436 ± 0.003	0.06 ± 0.01
Fresh chestnut wood	0.128 ± 0.003	0.19 ± 0.01
Chestnut tannins	0.019 ± 0.002	1.2 ± 0. 1
Exhausted quebracho wood	1.14 ± 0.04	0.051 ± 0.005
Fresh quebracho wood	0.0990 ± 0.0007	0.08 ± 0.01
Quebracho tannins	0.026 ± 0.002	0.47 ± 0.03
Trolox	0.015 ± 0.001	-

^1^ Reported are the mean ± SD values of at least three experiments. ^2^ EC_50_ is the dose of the material at which a 50% 2,2-diphenyl-1-picrylhydrazyl (DPPH) reduction is observed.

**Table 2:** Tannin content and total phenolic content (TPC) of wood samples.

Sample	Tannins (% *w*/*w*) ^1^	TPC (mg gallic acid/g of Sample) ^2^
Chestnut wood fiber	19	151 ± 17
Exhausted chestnut wood	11	51 ± 3
Fresh chestnut wood	21	153 ± 9
Chestnut tannins	100	457 ± 59
Exhausted quebracho wood	28	40 ± 1
Fresh quebracho wood	43	194 ± 8
Quebracho tannins	100	550 ± 99

^1^ Reported are the mean values of at least three experiments (SD ≤ 5%). ^2^ Reported are the mean values ± SD of at least three experiments.

**Table 3:** Activity of tannins and wood samples in the oxygen radical antioxidant capacity (ORAC) assay.^1^

Sample	Relative Fluorescence Intensity (%)
-	0.2 ^2^
Chestnut wood fiber	86
Exhausted chestnut wood	0.5
Fresh chestnut wood	89
Chestnut tannins	81
Exhausted quebracho wood	0.2
Fresh quebracho wood	90
Quebracho tannins	100

^1^ Reported are the mean values of at least three experiments (SD ≤ 5%). ^2^ Mixture containing only fluorescein and APPH, without any antioxidant.

**Table 4:** Pollutant adsorption properties of wood samples.^1^

Sample	MB Adsorption (%) ^2^	NO_x_ Scavenging (%) ^3^
Chestnut wood fiber	100	32 ± 5
Exhausted chestnut wood	73 ± 3	23 ± 1
Fresh chestnut wood	79 ± 1	38 + 1
Exhausted quebracho wood	77 ± 1	79 ± 1
Fresh quebracho wood	77 ± 2	86 ± 5

^1^ Reported are the mean ± SD values of at least three experiments. ^2^ Determined with methylene blue (MB) at 5 mg/L. ^3^ Determined with 0.6 mL of gas.

## References

[1] Panzella L., Napolitano A. (2017). Natural phenol polymers: Recent advances in food and health applications. Antioxidants.

[2] Piccinino D., Capecchi E., Botta L., Bizzarri B.M., Bollella P., Antiochia R., Saladino R. (2018). Layer-by-layer preparation of microcapsules and nanocapsules of mixed polyphenols with high antioxidant and UV-shielding properties. Biomacromolecules.

[3] Kurisawa M., Chung J.E., Uyama H., Kobayashi S. (2004). Oxidative coupling of epigallocatechin gallate amplifies antioxidant activity and inhibits xanthine oxidase activity. Chem. Commun..

[4] Panzella L., Cerruti P., Ambrogi V., Agustin-Salazar S., D’Errico G., Carfagna C., Goya L., Ramos S., Martín M.A., Napolitano A. (2016). A superior all-natural antioxidant biomaterial from spent coffee grounds for polymer stabilization, cell protection, and food lipid preservation. ACS Sustain. Chem. Eng..

[5] Panzella L., Pérez-Burillo S., Pastoriza S., Martín M.Á., Cerruti P., Goya L., Ramos S., Rufián-Henares J.Á., Napolitano A., d’Ischia M. (2017). High antioxidant action and prebiotic activity of hydrolyzed spent coffee grounds (HSCG) in a simulated digestion-fermentation model: Toward the development of a novel food supplement. J. Agric. Food Chem..

[6] Olejar K.J., Ray S., Ricci A., Kilmartin P.A. (2014). Superior antioxidant polymer films created through the incorporation of grape tannins in ethyl cellulose. Cellulose.

[7] Memoli S., Napolitano A., d’Ischia M., Misuraca G., Palumbo A., Prota G. (1997). Diffusible melanin-related metabolites are potent inhibitors of lipid peroxidation. Biochim. Biophys. Acta.

[8] Panzella L., D’Errico G., Vitiello G., Perfetti M., Alfieri M.L., Napolitano A., d’Ischia M. (2018). Disentangling structure-dependent antioxidant mechanisms in phenolic polymers by multiparametric EPR analysis. Chem. Commun..

[9] Ambrogi V., Panzella L., Persico P., Cerruti P., Lonz C.A., Carfagna C., Verotta L., Caneva E., Napolitano A., d’Ischia M. (2014). An antioxidant bioinspired phenolic polymer for efficient stabilization of polyethylene. Biomacromolecules.

[10] Ekambaram S.P., Perumal S.S., Balakrishnan A. (2016). Scope of hydrolysable tannins as possible antimicrobial agent. Phytother. Res..

[11] Freire M., Cofrades S., Perez-Jimenez J., Gomez-Estaca J., Jimenez-Colmenero F., Bou R. (2018). Emulsion gels containing n-3 fatty acids and condensed tannins designed as functional fat replacers. Food Res. Int..

[12] Cheaib D., El Darra N., Rajha H.N., El-Ghazzawi I., Mouneimne Y., Jammoul A., Maroun R.G., Louka N. (2018). Study of the selectivity and bioactivity of polyphenols using infrared assisted extraction from apricot pomace compared to conventional methods. Antioxidants.

[13] Smeriglio A., Barreca D., Bellocco E., Trombetta D. (2017). Proanthocyanidins and hydrolysable tannins: Occurrence, dietary intake and pharmacological effects. Br. J. Pharmacol..

[14] Hagerman A.E., Cheynier V., Sarni-Manchado P., Quideau S. (2012). Fifty years of polyphenol–protein complexes. Recent Advances in Polyphenol Research.

[15] Comandini P., Lerma-García M.J., Simó-Alfonso E.F., Toschi T.G. (2014). Tannin analysis of chestnut bark samples (*Castanea sativa* Mill.) by HPLC-DAD-MS. Food Chem..

[16] Reid D.G., Bonnet S.L., Kemp G., van der Westhuizen J.H. (2013). Analysis of commercial proanthocyanidins. Part 4: Solid state ^13^C NMR as a tool for in situ analysis of proanthocyanidin tannins, in heartwood and bark of quebracho and acacia, and related species. Phytochemistry.

[17] Redondo L.M., Chacana P.A., Dominguez J.E., Fernandez Miyakawa M.E. (2014). Perspectives in the use of tannins as alternative to antimicrobial growth promoter factors in poultry. Front. Microbiol..

[18] Cardullo N., Muccilli V., Saletti R., Giovando S., Tringali C. (2018). A mass spectrometry and ^1^H NMR study of hypoglycemic and antioxidant principles from a *Castanea sativa* tannin employed in oenology. Food Chem..

[19] Molino S., Fernandez-Miyakawa M., Giovando S., Rufian-Henares J.A. (2018). Study of antioxidant capacity and metabolization of quebracho and chestnut tannins through in vitro gastrointestinal digestion-fermentation. J. Funct. Foods.

[20] Poaty B., Dumarcay S., Gerardin P., Perrin D. (2010). Modification of grape seed and wood tannins to lipophilic antioxidant derivatives. Ind. Crops Prod..

[21] Aires A., Carvalho R., Saavedra M.J. (2016). Valorization of solid wastes from chestnut industry processing: Extraction and optimization of polyphenols, tannins and ellagitannins and its potential for adhesives, cosmetic and pharmaceutical industry. Waste Manag..

[22] Versari A., du Toit W., Parpinello G.P. (2013). Oenological tannins: A review. Aust. J. Grape Wine.

[23] Campo M., Pinelli P., Romani A. (2016). Hydrolyzable tannins from sweet chestnut fractions obtained by a sustainable and eco-friendly industrial process. Nat. Prod. Commun..

[24] Taflick T., Schwendler L.A., Rosa S.M.L., Bica C.I.D., Nachtigall S.M.B. (2017). Cellulose nanocrystals from acacia bark-Influence of solvent extraction. Int. J. Biol. Macromol..

[25] Panzella L., Eidenberger T., Napolitano A., d’Ischia M. (2012). Black sesame pigment: DPPH assay-guided purification, antioxidant/antinitrosating properties, and identification of a degradative structural marker. J. Agric. Food Chem..

[26] Verotta L., Panzella L., Antenucci S., Calvenzani V., Tomay F., Petroni K., Caneva E., Napolitano A. (2018). Fermented pomegranate wastes as sustainable source of ellagic acid: Antioxidant properties, anti-inflammatory action, and controlled release under simulated digestion conditions. Food Chem..

[27] Gökmen V., Serpen A., Fogliano V. (2009). Direct measurement of the total antioxidant capacity of foods: The ‘QUENCHER’ approach. Trends Food Sci. Technol..

[28] Goupy P., Dufour C., Loonis M., Dangles O. (2003). Quantitative kinetic analysis of hydrogen transfer reactions from dietary polyphenols to the DPPH radical. J. Agric. Food Chem..

[29] Benzie I.F.F., Strain J.J. (1996). The Ferric Reducing Ability of Plasma (FRAP) as a measure of “antioxidant power”: The FRAP assay. Anal. Biochem..

[30] Xu C., Liu S., Liu Z., Song F., Liu S. (2013). Superoxide generated by pyrogallol reduces highly water-soluble tetrazolium salt to produce a soluble formazan: A simple assay for measuring superoxide anion radical scavenging activities of biological and abiological samples. Anal. Chim. Acta.

[31] Drinkwater J.M., Tsao R., Liu R., Defelice C., Wolyn D.J. (2015). Effects of cooking on rutin and glutathione concentrations and antioxidant activity of green asparagus (*Asparagus officinalis*) spears. J. Funct. Foods.

[32] Panzella L., Melone L., Pezzella A., Rossi B., Pastori N., Perfetti M., D’Errico G., Punta C., d’Ischia M. (2016). Surface-functionalization of nanostructured cellulose aerogels by solid state eumelanin coating. Biomacromolecules.

[33] Panzella L., Manini P., Crescenzi O., Napolitano A., d’Ischia M. (2003). Nitrite-induced nitration pathways of retinoic acid, 5,6-epoxyretinoic acid, and their esters under mildly acidic conditions: Toward a reappraisal of retinoids as scavengers of reactive nitrogen species. Chem. Res. Toxicol..

[34] Manini P., Panzella L., Eidenberger T., Giarra A., Cerruti P., Trifuoggi M., Napolitano A. (2016). Efficient binding of heavy metals by black sesame pigment: Toward innovative dietary strategies to prevent bioaccumulation. J. Agric. Food Chem..

[35] Bosso A., Guaita M., Petrozziello M. (2016). Influence of solvents on the composition of condensed tannins in grape pomace seed extracts. Food Chem..

[36] Seifzadeha N., Saharia M.A., Barzegara M., Gavlighia H.A., Calanib L., Del Rio D., Galaverna G. (2019). Evaluation of polyphenolic compounds in membrane concentrated pistachio hull extract. Food Chem..

[37] Peng Y., Liu R., Cao J. (2015). Characterization of surface chemistry and crystallization behavior of polypropylene composites reinforced with wood flour, cellulose, and lignin during accelerated weathering. Appl. Surf. Sci..

[38] Neagu R.C., Cuénoud M., Berthold F., Bourban P.E., Gamstedt E.K., Lindström M., Månson J.A.E. (2012). The potential of wood fibers as reinforcement in cellular biopolymers. J. Cell. Plast..

[39] Gajewski M., Kowalczyk K., Bajer M. (2010). Influence of ecological friendly mediums on chemical composition of greenhouse-grown eggplants. Ecol. Chem. Eng. A.

[40] Panzella L., Manini P., Napolitano A., d’Ischia M. (2005). The acid-promoted reaction of the green tea polyphenol epigallocatechin gallate with nitrite ions. Chem. Res. Toxicol..

