# Correction: Exhausted Woods from Tannin Extraction as an Unexplored Waste Biomass: Evaluation of the Antioxidant and Pollutant Adsorption Properties and Activating Effects of Hydrolytic Treatments. *Antioxidants* 2019, *8*, 84

**DOI:** 10.3390/antiox8060157

**Published:** 2019-06-03

**Authors:** Lucia Panzella, Federica Moccia, Maria Toscanesi, Marco Trifuoggi, Samuele Giovando, Alessandra Napolitano

**Affiliations:** 1 Department of Chemical Sciences, University of Naples “Federico II”, Via Cintia 4, I-80126 Naples, Italy; federica.moccia@unina.it (F.M.); maria.toscanesi@unina.it (M.T.); marco.trifuoggi@unina.it (M.T.); alesnapo@unina.it (A.N.); 2 Centro Ricerche per la Chimica Fine Srl for Silvateam Spa, Via Torre 7, 12080 San Michele Mondovì, CN, Italy; sgiovando@silvateam.com

The authors wish to make the following corrections to their paper [1]. The authors wish to insert Acknowledgments section: 

The authors wish to thank the European Union (FSE, PON Ricerca e Innovazione 2014-2020, Azione I.1 “Dottorati Innovativi con caratterizzazione Industriale”) for funding a PhD grant to Federica Moccia.

The authors would like to apologize for any inconvenience caused. The change does not affect the scientific results. The manuscript will be updated and the original will remain online on the article webpage.
